# Preload Monitoring in Bolted Connection Using Piezoelectric-Based Smart Interface

**DOI:** 10.3390/s18092766

**Published:** 2018-08-22

**Authors:** Thanh-Canh Huynh, Ngoc-Loi Dang, Jeong-Tae Kim

**Affiliations:** Department of Ocean Engineering, Pukyong National University, Busan 48723, Korea; ce.huynh@gmail.com (T.-C.H.); loi.ngocdang@gmail.com (N.-L.D.)

**Keywords:** preload monitoring, bolted connection, bolt-loosening, piezoelectric sensor, impedance response, smart interface

## Abstract

In this study, a preload monitoring method using impedance signatures obtained from a piezoelectric-based smart interface is presented for bolted girder connections. Firstly, the background theory of the piezoelectric-based smart interface and its implementation into the health monitoring of bolted connections are outlined. A simplified electro-mechanical (EM) impedance model of a smart interface-embedded bolted connection system is formulated to interpret a mechanistic understanding of the EM impedance signatures under the effect of bolt preload. Secondly, finite element modeling of a bolted connection is carried out to show the numerical feasibility of the presented method, and to predetermine the sensitive frequency band of the impedance signatures. Finally, impedance measurements are conducted on a lab-scaled bolted girder connection, to verify the predetermined sensitive frequency range and to assess the bolt preload changes in the test structure.

## 1. Introduction

Bolting is a widely-accepted method for making connections in steel structures in the field. Bolts are torqued to a high tensile stress, developing clamping pressures at the interfaces of the structural members to hold them in position. Followed by the use of high-strength bolts, this fastening method has enabled the advantages of easy installation, time efficiency, and a high strength for field connections. After a long-term service life, however, the bolted connections could experience a loss of preloads (i.e., self-loosening) due to repetitive external forces and vibrations, which threaten their functionality. Therefore, bolt preload monitoring is essential and recently gained growing interest in efforts to ensure the safety of bolted joints, and to prevent the catastrophic failures of the entire structures [1,2,3,4,5,6].

To assess the structural integrity of the local critical members in the mechanical and civil systems, there have been many research attempts on the impedance-based method [7,8,9,10,11,12,13]. The fundamental part of the method is to utilize electromechanical (EM) impedance responses as local dynamic features for assessing the structural damage. The frequency band used in the impedance-based method is often in the ultrasonic range, hence the method is able to effectively capture incipient damages. Owing to the advantage associated with the use of high-frequency responses, the impedance-based method has been applied for the health assessment of bolted joints [8,14,15,16,17,18].

Bolt-loosening in a bolted joint can be monitored via its EM impedance responses, measured by piezoelectric sensors or piezoelectric washers [15,18,19,20,21,22]. Because the EM impedance correlates with the structural properties of a bolted connection, any damage occurrence could be detected via observing the changes in measured impedance data. From the previous research attempts on the impedance-based bolt-loosening monitoring, an important question has been raised on how to identify an effective frequency band of impedance signatures that is sensitive to the preload change or bolt-loosening. In real situations, the effective frequency band is often determined by trial-and-error, because it is dependent on the local dynamic characteristics of a monitored structure.

The mountable interface technique can be a potential solution to cope with the above-mentioned problem [23]. This technique uses an interfacial structure equipped with a piezoelectric sensor (e.g., PZT (lead zirconate titanate)) to indirectly acquire the sensitive impedance data from a target structure. The geometry and material properties of the interface should be appropriately designed so that the sensitive impedance response is occurred within a pre-defined frequency band. For the damage monitoring of bolted joints, the mountable interface technique could offer unique advantages in comparison with the piezoelectric washer technique [20,21,22]. Firstly, the mountable interface can be post-installed into an existing connection, whereas the piezoelectric washer requires pre-installation during the construction. Secondly, a single mountable interface can be used to monitor multiple bolts in a connection, meanwhile, a single piezoelectric washer is particularly fit with a single bolt. Thus, the use of the mountable interface technique could reduce the number of sensing channels for impedance monitoring of a large bolted connection. However, the previous studies have mainly focused on developing the mountable interface technique for the health monitoring of tendon-anchorage systems [23,24,25,26]. So far, the effectiveness of the mountable interface technique for bolt-loosening detection problems has not been evaluated. Additionally, the mechanistic understanding of the impedance response under bolt-loosening has not been sufficiently explained via a mathematical model that considers the effect of a bolted connection. Also, there is a need to identify the sensitive frequency band for the impedance monitoring of a bolted connection by using finite element modeling.

In this study, a PZT interface-based impedance monitoring method is developed to detect the bolt-loosening events in a bolted connection. To demonstrate the theoretical feasibility of the presented method, a simplified impedance model is newly designed with the consideration of the bolt preload effect. Next, the sensitive frequency band of the EM impedance responses is numerically predetermined for a bolted connection example embedded with a PZT interface. Finally, impedance measurements are conducted on a lab-scaled bolted girder connection to verify the pre-analyzed sensitive frequency band and to evaluate the effectiveness of the proposed method for bolt-loosening detection. 

## 2. Piezoelectric-Based Smart Interface for Bolted Connection

### 2.1. Piezoelectric-Based Smart Interface Technique

**Figure 1:**
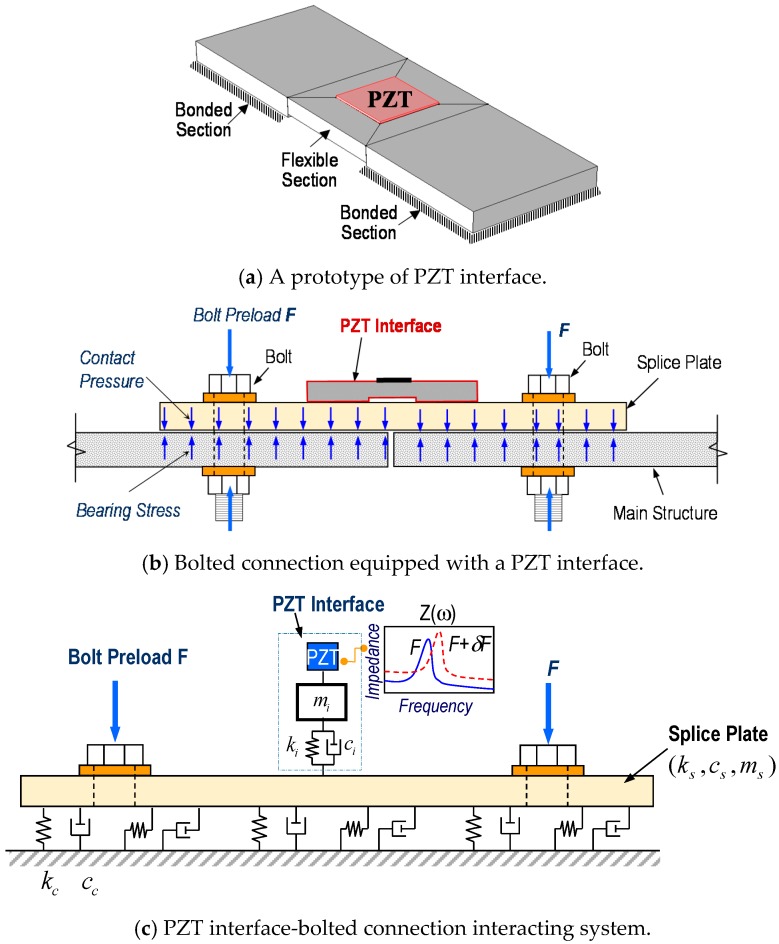
Impedance monitoring method for bolted connection via PZT interface.

An impedance monitoring method using the PZT interface technique is designed in order to acquire the impedance data with predetermined sensitive frequency bands from bolted connections. As shown in Figure 1a, the PZT interface prototype is a plate-like structure, having two outside bonded sections and a middle flexible section, that is embedded with a PZT sensor. The flexible section is intentionally made to provide free vibrations during the PZT’s excitation. The bonded sections allow the PZT interface prototype to be mountable and easily reconfigured if needed. 

To monitor multiple bolts in a connection, the PZT interface should be mounted to the splice plate connection, which is clamped by the bolt preloads, as shown in Figure 1b. Under the PZT’s excitation, there are coupled interactions between the PZT and the interface, and then between the PZT interface and the connection splice plate. The coupling between the PZT interface and the splice plate opens a potentiality to assess multiple loosened bolts on the splice plate. The flexible section of the PZT interface allows for predetermining the sensitive frequency band of impedance signals below 100 kHz, and thus enabling the use of a low-cost wireless impedance measurement system [22,27,28].

In equilibrium, the bolt preloads can be transformed into contact pressures and bearing stresses at the contact between the main structure and the splice plate, as seen in Figure 1b. According to the previous studies [17,29], the contact parameters of the bolted connection can be represented by a system of a spring and dashpot (*k_c_*, *c_c_*), whose values represent the amount of bolt preloads, see Figure 1c. At the PZT driving point, the interface can be modeled with the mass, stiffness, and damping parameters (*m_i_*, *k_i_*, and *c_i_*) and the splice plate can be also modeled by the respectively structural parameters (*m_s_*, *k_s_*, and *c_s_*). When the bolt preloads are changed, the contact parameters of the connection are altered (e.g., contact stiffness reduction), leading to the variation in the coupled responses of the system at resonance. By monitoring the impedance responses of the system in the resonant band, it is possible to detect the bolt looseness or preload changes that have occurred in the connection.

### 2.2. Analytical Modeling of Piezoelectric-Based Smart Interface

#### 2.2.1. Impedance Response of Bolted Connection

**Figure 2:**
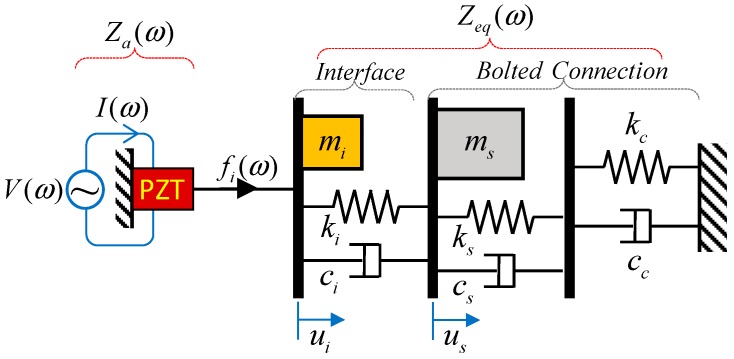
Impedance model of the PZT interface-bolted connection system.

The impedance responses of a bolted connection measured via the PZT interface can be theoretically derived from a simplified impedance model. Based on the previous studies [10,30], a two-dof (degree of freedom) impedance model with the consideration of the contact parameters representing the bolt preload is proposed, as shown in Figure 2. In the model, one dof refers to the interface (*m_i_*, *c_i_*, and *k_i_*), and the other dof refers to the splice plate (*m_s_, c_s_*, and *k_s_*) with the contact parameters (*c_c_* and *k_c_*).

As illustrated in Figure 2, when the PZT sensor is excited by a harmonic voltage, *V*(*ω*), with a current, *I*(*ω*), a harmonic force is introduced into the system at the PZT driving point. The equation of motion under the PZT’s harmonic force $f_{i}=F_{i}e^{j\omega t}$ can be given as follows:

(1) $$\begin{array}{r} m_{i}{\ddot{u}}_{i}+c_{i}({\dot{u}}_{i}-{\dot{u}}_{s})+k_{i}(u_{i}-u_{s})=f_{i}\\ m_{s}{\ddot{u}}_{s}+\frac{c_{s}c_{c}\;}{c_{s}+c_{c}}{\dot{u}}_{s}-c_{i}({\dot{u}}_{i}-{\dot{u}}_{s})+\frac{k_{s}k_{c}}{k_{s}+k_{c}}u_{s}-k_{i}(u_{i}-u_{s})=0 \end{array}$$

where $u_{i},\dot{u_{i}},\;{\ddot{u}}_{i}$ and $u_{s},\dot{u_{s}},{\ddot{u}}_{s}$ are the displacements, velocities, and accelerations corresponding to masses *m_i_* and *m_s_*, respectively. 

Under the harmonic excitation force, the steady states of the interface and the splice plate can be described by the following:

(2) $$\begin{array}{l} u_{i}=U_{i}e^{j\omega t\;}\\ u_{s}=U_{s}e^{j\omega t} \end{array}$$

where *U_i_* and *U_s_* are complex quantities that are dependent on the excited frequency and structural parameters of the system. By substituting Equation (2) into Equation (1), equations to obtain *U_i_* and *U_s_* of the system are given as follows:

(3) $$\begin{array}{r} \left(-\omega^{2}m_{i}+j\omega c_{i}+k_{i}\;\right)U_{i}-\left(j\omega c_{i}+k_{i}\right)U_{s}=F_{i}\\ -\left(j\omega c_{i}+k_{i}\right)U_{i}+\left(-\omega^{2}m_{s}+j\omega (c_{i}+\frac{c_{s}c_{c}}{c_{s}+c_{c}})+(k_{i}+\frac{k_{s}k_{c}}{k_{s}+k_{c}})\right)U_{s}=0 \end{array}$$

The equivalent mechanical impedance of the system, *Z_eq_*, is defined as the ratio between the excitation force *f_i_* and the velocity at the PZT driving point $\dot{u_{i}}$, given as follows:

(4) $$Z_{eq}=\frac{f_{i}}{{\dot{u}}_{i}}=\frac{F_{i}e^{j\omega t}}{j\omega U_{i}e^{j\omega t}}$$

By solving Equation (3), the quantity, *U_i_*, is obtained. By substituting the obtained *U_i_* into Equation (4), the equivalent mechanical impedance of the system *Z_eq_* is obtained as follows:

(5) $$Z_{eq}=\frac{\left(-\omega^{2}m_{i}+j\omega c_{i}+k_{i}\right)\left(-\omega^{2}m_{s}+j\omega (c_{i}+\frac{c_{s}c_{c}}{c_{s}+c_{c}})+(k_{i}+k_{s}\frac{1}{1+\xi })\right)-{\left(j\omega c_{i}+k_{i}\right)}^{2}}{j\omega \left(-\omega^{2}m_{s}+j\omega (c_{i}+\frac{c_{s}c_{c}}{c_{s}+c_{c}})+(k_{i}+k_{s}\frac{1}{1+\xi })\right)}$$

where *ξ = k_s_/k_c_* is defined as the ratio between the splice plate’s stiffness and the contact stiffness. *ξ* ≈ 0 indicates the infinitive value of contact stiffness (i.e., fixed boundary), while *ξ* ≈ ∞ indicates the unnoticeable value of the contact stiffness (i.e., free boundary). If the splice plate’s stiffness, *k_s_*, remains unchanged, the increment of the ratio *ξ = k_s_/k_c_* will be equivalent to the decrement of the contact stiffness, *k_c_*, which can be interpreted as the bolt preload reduction (i.e., bolt looseness).

The EM impedance, *Z*(*ω*), of the bolted connection measured via the PZT interface is a combined function of the equivalent mechanical impedance of the interface-bolted connection system, *Z_eq_*, and the mechanical impedance of the PZT sensor, *Z_a_*, given by [30,31] the following:

(6) $$Z(\omega )={\left\{j\omega \frac{w_{a}l_{a}}{t_{a}}\left[\varepsilon_{33}^{T}(1-j\delta )-d_{31}^{2}{\hat{Y}}_{11}^{E}+\frac{Z_{a}(\omega )}{Z_{a}(\omega )+Z_{eq}(\omega )}d_{31}^{2}{\hat{Y}}_{11}^{E}\frac{\tan(kl_{a})}{kl_{a}}\right]\right\}}^{-1}$$

where ${\hat{Y}}_{11}^{E}=\left(1+j\eta \right)Y_{11}^{E}$ is the complex Young’s modulus of the PZT sensor (width $w_{a}$, length $l_{a}$, and thickness $t_{a}$) at the zero electric field; $\varepsilon_{33}^{T}$ is the dielectric constant at the zero stress; $d_{31}$ is the piezoelectric coupling constant in the 1-direction at the zero stress; the terms *η* and *δ* are the structural damping loss factor and the dielectric loss factor of the PZT. The wave number of the PZT is given as $k=\omega \sqrt{\rho /Y_{11}^{E}}$, where *ρ* is the mass density of the PZT. The mechanical impedance of the PZT is computed as $Z_{a}=-j{\hat{Y}}_{11}^{E}w_{a}t_{a}/\omega l_{a}$.

From Equations (5) and (6), it has been shown that the impedance response, *Z*(*ω*), measured via the PZT interface, would contain the structural parameters of the interface and the connection. Thus, any damage that occurred in the bolted connection (e.g., preload change) can be diagnosed by tracking the variation in the impedance response, *Z*(*ω*).

#### 2.2.2. Impedance Response versus Preload Change

To demonstrate the theoretical feasibility of the PZT interface technique for the impedance monitoring of a bolted connection, an example of the simplified two-dof model was investigated. The PZT sensor has the following dimensions: $w_{a}$ = 15 mm, $l_{a}$ = 15 mm, and $t_{a}$ = 0.51 mm; and the following properties: *ρ* = 7750 kg/m^3^, $Y_{11}^{E}$ = 6.098 × 10^10^ N/m^2^, $\varepsilon_{33}^{T}$ = 1.505 × 10^−8^ Farads/m, $d_{31}$ = −1.71 × 10^10^ m/V, *δ* = 0.015, and *η* = 0.0125. The interface has the following structural properties: *m_i_* = 0.1 kg, *k_i_* = 2 × 10^9^ N/m, *c_i_* = 200 N/ms^−1^; and the connection splice plate has the following structural properties: *m_s_* = 1 kg, *k_s_* = 2 × 10^10^ N/m, *c_s_* = 200 N/ms^−1^. The contact damping is assumed as *c_c_* = 500 N/ms^−1^. Assuming that the splice plate was undamaged (i.e., *k_s_* remained constant), the bolt preload reduction can be simulated by increasing the stiffness ratio *ξ = k_s_/k_c_* (i.e., *k_c_* was reduced with respect to *k_s_*).

**Figure 3:**
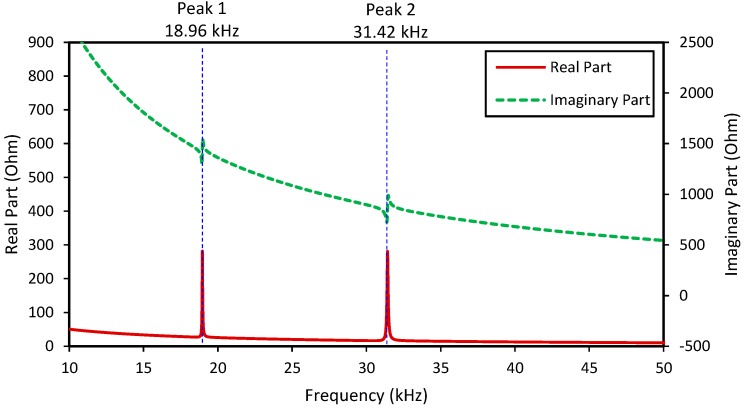
Impedance responses of the impedance model with infinitive contact stiffness (*ξ = k_s_/k_c_* = 0).

Figure 3 shows the real and imaginary impedance responses, *Z*(*ω*), of the two-dof model when the contact stiffness was infinitive (*ξ* = *k_s_/k_c_* = *k_s_/*∞ = 0). Two resonant peaks (i.e., Peak 1 at 18.96 kHz and Peak 2 at 31.42 kHz) can be clearly observed from the impedance responses, representing the two coupling responses of the system. It is noted that the resonant impedance peaks represent the significant contributions of the equivalent structural impedance, *Z_eq_*, to the total impedance *Z*(*ω*) (see Equation (6)).

**Figure 4:**
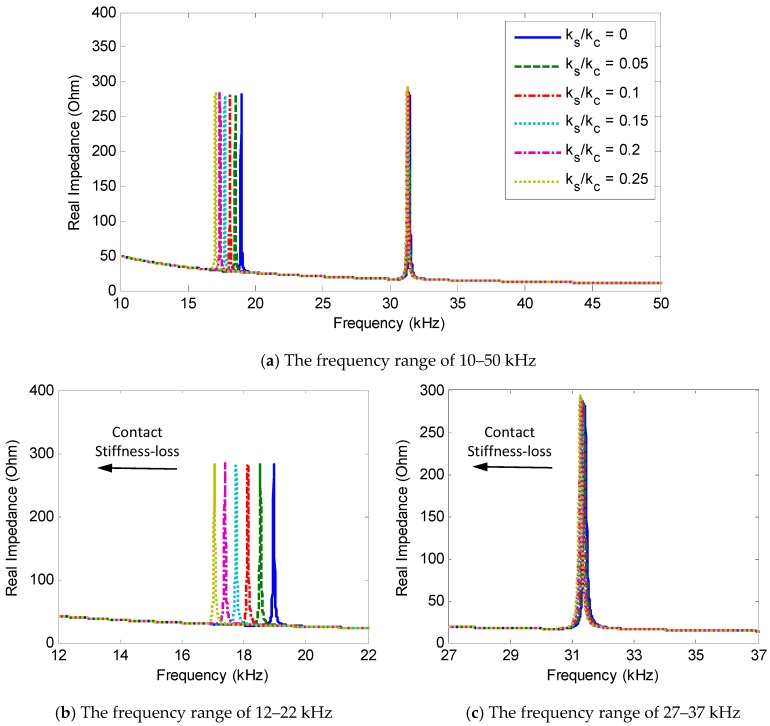
Changes in the impedance responses of the impedance model under contact stiffness-loss.

The effect of the bolt preload reduction on the impedance responses was investigated for the different stiffness ratio, *k_s_/k_c_*, in the range of 0–0.25, as plotted in Figure 4a. The changes in the real impedance values of the two impedance peaks were zoomed in Figure 4b,c, respectively. From the figures, it can be seen that as the ratio, *k_s_/k_c_*, was increased from 0 to 0.25 (i.e., the contact stiffness, *k_c_ˆ,* was reduced), the two resonant peaks clearly shifted to the left side, indicating the reduction in the resonant frequencies of the system.

When the ratio, *k_s_/k_c_*, was varied from 0 to 0.25, Peak 1′s frequency was varied from 18.96 kHz to 17.06 kHz (i.e., 10.02% variation) and Peak 2′s frequency was shifted from 31.42 kHz to 31.25 kHz (i.e., 0.54% variation). The results suggested that the impedance peak at a lower frequency exhibited a larger frequency shift than that at a higher frequency under the same bolt preload change. Importantly, the results evidenced the theoretical feasibility of the PZT interface technique for the bolt-loosening monitoring of a bolted connection.

## 3. Predetermination of Sensitive Frequency Band for Impedance Response

### 3.1. Finite Element Model of PZT Interface-Bolted Connection

#### 3.1.1. Finite Element Modeling

**Figure 5:**
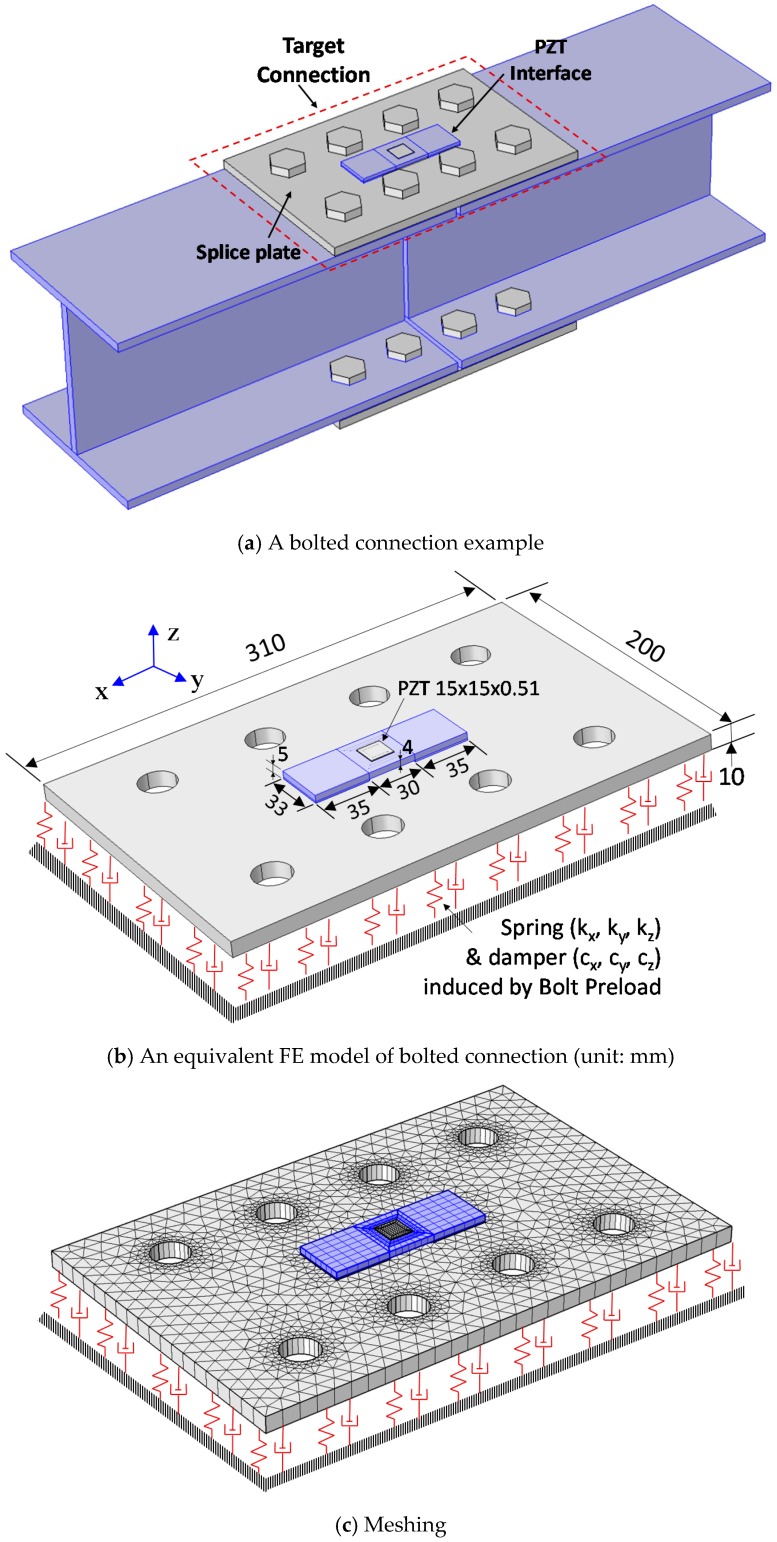
A finite element (FE) modeling of a bolted connection example with a PZT interface.

The EM impedance’s sensitive frequency band was predetermined for a bolted connection by using the PZT interface technique. The finite element (FE) model of a bolted connection example was established by using COMSOL multiphysics. As shown in Figure 5a, the connection example is a steel bolted joint that was used to connect two H-beam segments. The splice plate (310 × 200 × 10 mm^3^) was clamped by the eight bolts (20 mm diameter) at each flange of the H-beam. To monitor the bolt preload, the connection was equipped with a PZT interface at the middle of the splice plate. The effect of the bolt preload was simulated by the equivalent contact spring (*k_x_*, *k_y_*, and *k_z_*) and the damper systems (*c_x_*, *c_y_*, and *c_z_*), as shown in Figure 5b [17,30]. The main concern of the FE study was to numerically examine the effect of the contact parameters on the impedance responses of the PZT interface. So, the effect of the H-beam segments in Figure 5a was neglected for the simplification. 

As detailed in Figure 5b, the PZT interface has two bonded sections (33 × 35 × 5 mm^3^) and a flexible section (33 × 30 × 4 mm^3^) embedded with a PZT-5A patch (15 × 15 × 0.51 mm^3^, Piezo Systems Inc). The interface body is made of aluminium. The PZT patch was attached to the interface by the bonding layer of 0.1 mm. The PZT interface was also mounted to the splice plate by the 0.1 mm bonding layer. The PZT patch was simulated using the piezoelectric elements that have both mechanical and electrical properties. The FE model was discretized by three-dimensional (3D) solid elements, as shown in Figure 5c. A complete mesh of the FE model consists of 4155 elements.

The structural properties of the splice plate, the interface, and the bonding layers are listed in Table 1. It is noted that the similar structural parameters of the bonding layers were recommended in the previous studies [32,33]. The piezoelectric properties of the PZT-5A are listed in Table 2 [26]. The thickness frequency of the PZT patch is about 4 MHz. For acquiring the EM impedance from the PZT interface, the harmonic excitation voltage with 1 V amplitude ($V=1e^{j\omega t}$) was applied to the top surface of the PZT sensor, while the bottom surface was set as the ground.

#### 3.1.2. Simulation of Bolt Preload Change

As explained previously, the bolt preload change can be represented by the variation of the contact parameters. The contact stiffness was assumed to be uniform over the contact area of the splice plate. For the intact state, the contact stiffness was set as *k_z_* = 4.0 × 10^11^ N/m/m^2^ and *k_x_* = *k_y_* = 0.5 *k_z_*. The contact damping loss factor was assumed to be *η_x_ = η_y_ = η_z_* = 0.02. As given in Table 3, four damage cases of the contact stiffness (D1–D4) were investigated. The contact stiffness was reduced by 12.5%, 25%, 37.5%, and 50% in the cases D1, D2, D3, and D4, respectively. It is noted that the contact stiffness-loss of 12.5% could be interpreted as the equivalent damage severity of a completely loosened bolt in the eight-bolt connection.

### 3.2. Predetermination of Sensitive Frequency Band for Bolted Connection

**Figure 6:**
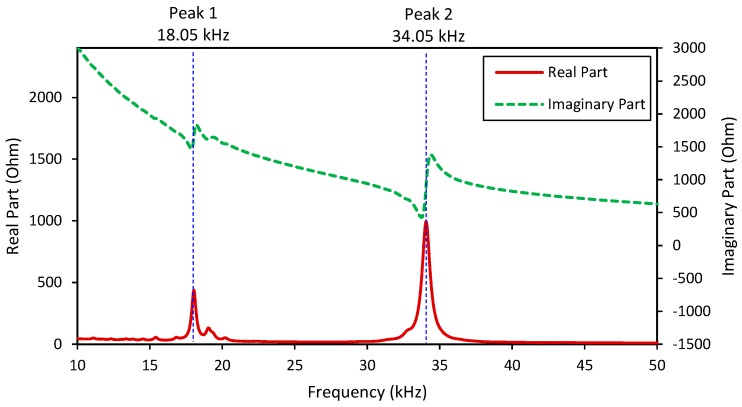
Numerical impedance signatures of PZT interface-bolted connection system.

Figure 6 shows the EM impedance of the PZT interface-bolted connection system, including the real and imaginary parts in the frequency range of 10–50 kHz with the resolution of 0.05 kHz. Within the examined range, there were both resonant and non-resonant regions of the impedance signatures. Two resonant bands containing two significant peaks (i.e., Peak 1 at 18.05 kHz and Peak 2 at 34.05 kHz) were observed in the figure. In the resonant bands, the aspect of the real impedance values becomes significant as that of the imaginary impedance values. Because the impedance signatures of Peaks 1–2 would be sensitive to structural damage, it is necessary to predetermine the frequency ranges containing these peaks.

**Figure 7:**
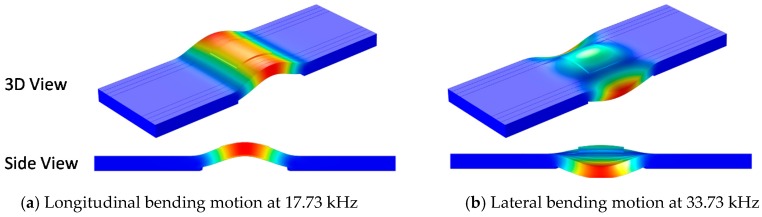
Two bending modes of PZT interface corresponding to Peak 1 and Peak 2.

To identify the modal responses corresponding to the two impedance peaks, the Eigenvalue analysis of the PZT interface was performed. The interface was fixed at the bottom surfaces of two bonded sections. As shown in Figure 7a,b, the bending modes of the PZT interface corresponding to Peak 1 and Peak 2 were found at the frequencies of 17.73 kHz (i.e., longitudinal bending motion) and 33.73 kHz (i.e., lateral bending motion), respectively. The frequency differences between the impedance analysis and the modal analysis of the isolated PZT interface were only 5.4% for Peak 1 and 0.9% for Peak 2. The results suggested that the sensitive frequency bands of the impedance signatures can be easily predetermined by the numerical modal analysis of the isolated PZT interface. The results also revealed that at least two significant peaks (Peaks 1–2) can be expected in the frequency band of 10–50 kHz for the impedance measurement via the PZT interface.

### 3.3. Evaluation of Predetermined Frequency Band

**Figure 8:**
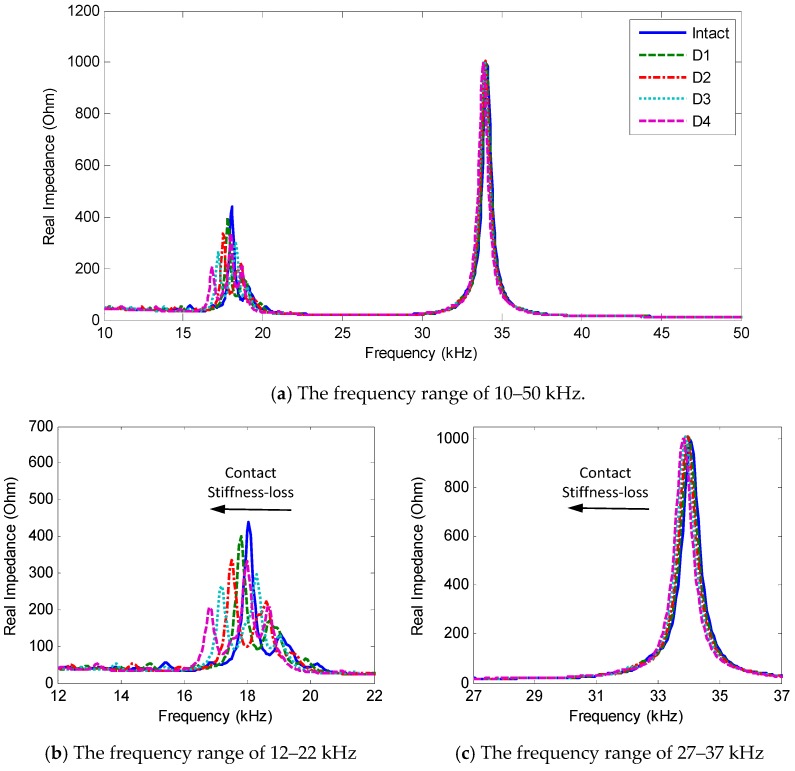
Numerical impedance signatures of FE model under contact stiffness-loss.

The sensitivity of the predetermined frequency band to the bolt preload change was numerically evaluated. The impedance signatures in 10–50 kHz were numerically analyzed for the four damage cases, as plotted in Figure 8a. Two resonant bands containing Peak 1 and Peak 2 were zoomed in Figure 8b,c, respectively. These impedance peaks sensitively shifted leftward along with the reduction in the contact stiffness. While Peak 1 in 12–22 kHz (see Figure 8b) experienced both the frequency and magnitude shifts, Peak 2 in 27–37 kHz (see Figure 8c) showed only the frequency variation. 

It is shown that Peak 1 was more sensitive to the bolt looseness than Peak 2. As listed in Table 4, when the contact stiffness was reduced by 50%, Peak 1′s frequency shifted 1.25 kHz (i.e., 6.93% variation), while Peak 2′s frequency shifted only 0.2 kHz (i.e., 0.59% variation). These results were well consistent with the previous observations from the two-dof impedance model, and also demonstrated the sensitivity of the PZT interface’s predetermined frequency range to the bolt preload change.

## 4. Experimental Evaluation on Lab-Scaled Bolted Girder Connection

### 4.1. Experimental Setup

#### 4.1.1. Test-Setup of Bolted Girder Connection

**Figure 9:**
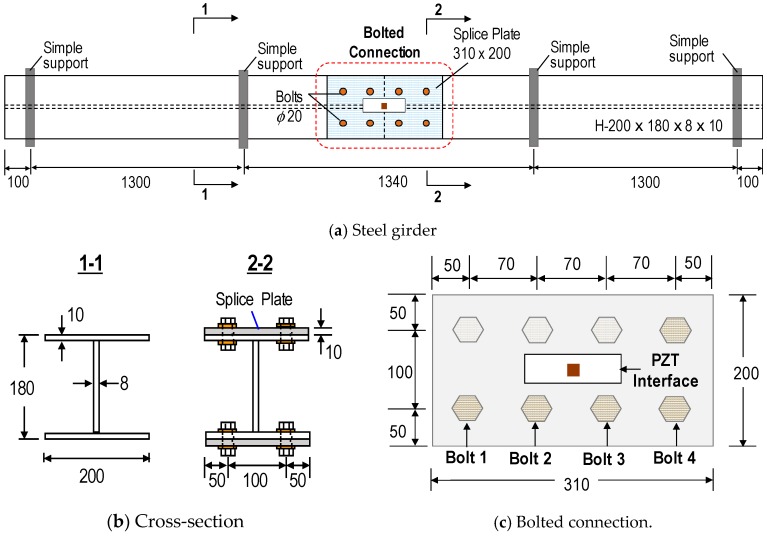
Schematic of the bolted girder connection (unit: mm).

An experimental evaluation was conducted on a lab-scaled bolted girder connection. Figure 9 shows the schematic of a three-span steel girder with a bolted connection at the middle. The girder, with a total length of 4.14 m, was simply supported by steel bars at four locations, as shown in Figure 9a. The girder was assembled from two single H-shaped beams (H-200 × 180 × 8 × 10 mm) by splice plates and bolts at two flanges, see Figure 9b. The connection splice plate (310 × 200 × 10 mm) clamped by eight bolts (20 mm-diameter) is schematized in Figure 9c. The PZT interface prototype sketched in Figure 5b was fabricated and surface-mounted to the middle of the splice plate. The whole body of the interface, including the flexible and side sections, was fabricated from an aluminium plate using a precision cutting machine. Loctite 401 instant adhesive was used to attach the PZT to the middle section and the bonded sections to the host structure.

**Figure 10:**
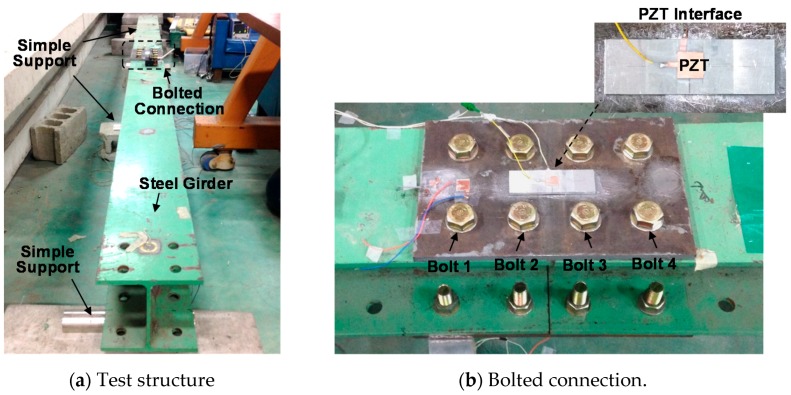
Experimental setup of bolted girder connection.

The real setups of the steel girder and the bolted connection are illustrated in Figure 10. As designed, all of the bolts of the connection were fastened to the torque of 160 Nm. A torque wrench (TOHNICHI QL280N) was used to fasten the bolts and to control the bolt torque. Four of the eight bolts in the connection (Bolts 1–4) were selected to simulate the loosening events, as indicated in Figure 10b. Among the four bolts, Bolts 1 and 3 are close to the PZT interface, while Bolts 1 and 4 are more distant. Table 5 describes the loosening cases of Bolt 1–4. Each of the four bolts was loosened from the initial torque of 160 Nm to the torque of 110 Nm (i.e., a 31% torque-loss), 60 Nm (i.e., a 62% torque-loss), and 0 Nm (i.e., a 100% torque-loss). The girder was placed in the laboratory, where the temperature was controlled near 22 °C by air-conditioners so as to avoid temperature effects.

#### 4.1.2. Impedance Measurement System

**Figure 11:**
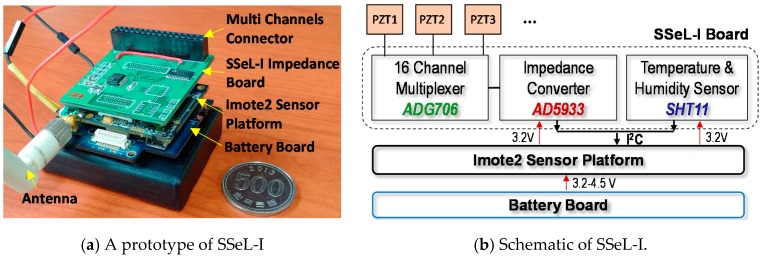
Wireless SSeL-I impedance sensor node.

A low-cost and multi-channel SSeL-I impedance measurement system developed by the research group at Pukyong National University [22], was used to wirelessly acquire the impedance data from the PZT interface. Figure 11a shows a prototype of the wireless SSeL-I node that consists of three layers, an SSeL-I impedance board, an Imote2 platform, and a battery board. The schematic of the SSeL-I sensor node is shown in Figure 11b. The key component of the SSeL-I board is the low-cost impedance chip AD9533, which has a capability to measure the impedance up to 100 kHz with the resolution less than 0.1 Hz. The SSeL-I board integrates a multiplexer for measuring up to 16 PZT patches and the SHT11 sensor for recording temperature and humidity. 

The Imote2 platform is used to control impedance measurements via the impedance board. The Imote2 has a high-speed PXA27x processor (clock speed of 13-416 MHz), SRAM of 256 kB, the flash memory of 32 MB, and the SDRAM of 32 MB [34,35,36]. This platform is designed with a wireless radio of 2.4 GHz Zigbee for data transmission (up to a distance of 125 m by an external antenna). The wireless sensor unit is powered via the battery board (3.2 V). Although the wireless impedance sensor node has a limited measurable frequency range (i.e., less than 100 kHz), it costs only 300 USD and has multi-channels that could enable the cost-effectiveness for a health monitoring system of in situ mega bolted structures. 

### 4.2. Preload Change Monitoring in Bolted Girder Connection

#### 4.2.1. Impedance Measurement via PZT Interface

**Figure 12:**
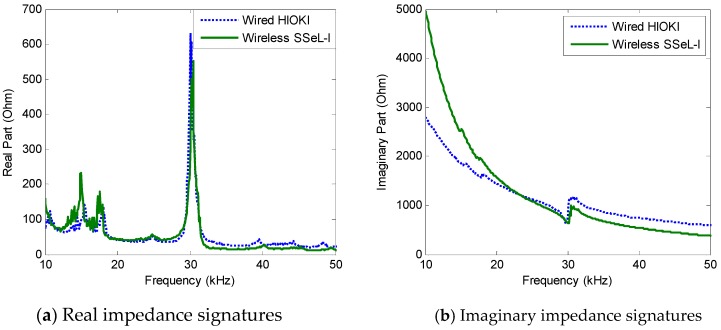
Impedance signatures in 10–50 kHz: wired versus wireless measurements.

The impedance signatures were measured in the frequency range of 10–50 kHz to identify the sensitive impedance peaks (Peaks 1–2), as numerically pre-analyzed in Section 3. The amplitude of the excitation voltage was set at 1V, and the resolution of the PZT scanning frequency was 0.1 kHz. Four repeated measurements were conducted for each of the bolt-loosening cases in Table 5. For the performance evaluation, the impedance signatures measured by the wireless SSeL-I system were compared with those using a wired high-performance impedance analyzer HIOKI-3532. As shown in Figure 12a,b, the real and imaginary impedance signatures measured by the SSeL-I system were well-matched with those by the wired HIOKI system for the same frequency range with identical patterns. 

**Figure 13:**
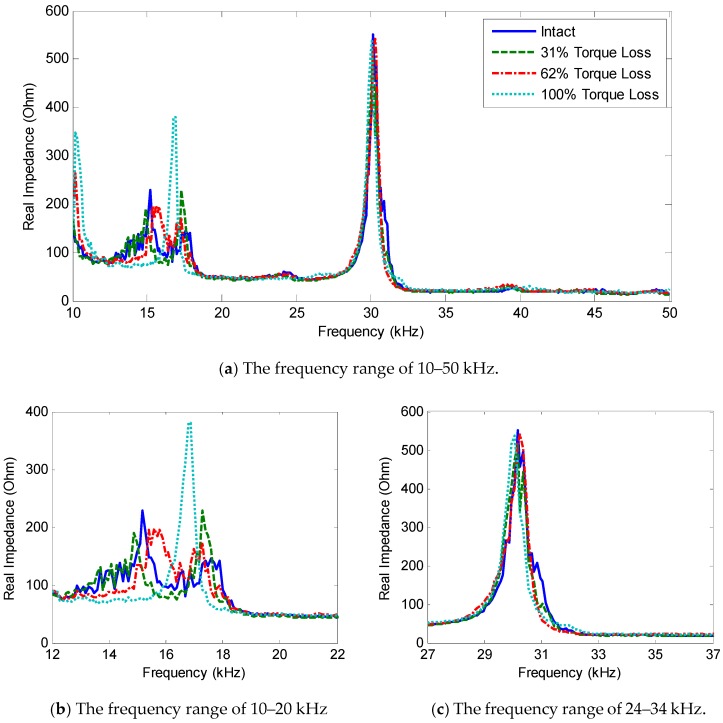
Experimental impedance signatures under bolt-loosening cases of Bolt 3.

The impedance responses under the bolt-loosening cases of Bolt 3 were plotted in Figure 13. As expected from the numerical analysis, two resonant bands (i.e., 12–22 kHz and 27–37 Hz) exist containing significant impedance peaks between 10–50 kHz, as zoomed in Figure 13b,c. The comparison between Figure 13 and Figure 8 revealed certain gaps between the experimental measurement and the numerical analysis. These gaps could be caused by the differences in the structural parameters between the experimental model and the FE model. For Peak 1, the numerical pre-analysis in Figure 8b predicted the peak frequencies around 18 kHz, while the experimental measurement in Figure 13b showed the peak frequencies near 16 kHz (i.e., the prediction error of 11.1%). For Peak 2, the pre-analysis in Figure 8c estimated the peak frequency at about 34 kHz, while the experiment in Figure 13c measured the peak frequency at about 30 kHz (i.e., the prediction error of 11.8%).

As observed in Figure 13, the impedance peaks tended to shift left as the torque was reduced. As compared with the first resonant band (i.e., 12–22 kHz), the second one (i.e., 27–37 kHz) showed less sensitivity to the torque-loss severity. The changing trend of the two resonant bands was consistent with the previous numerical results. The first resonant band experienced both the frequency and magnitude shifts, while the second one showed slight changes in the peak frequency and almost no noticeable changes in the magnitude.

#### 4.2.2. Detection of Preload Change using Impedance Response

##### Statistical Quantification Method

To detect the preload change in the bolted connection, two common damage-sensitive features were extracted from the impedance data, the correlation coefficient deviation (CCD) and root-mean-square deviation (RMSD) indices. These two impedance features quantify the changes in the impedance signatures with different manners. While the CCD index mainly quantifies the frequency shift of the impedance signatures, the RMSD index quantifies both the frequency and magnitude shifts. 

According to [23], the CCD index can be computed using the below formula:

(7) CCD=1−1σZσZ*E{[Re(Z(ωi))−Re(Z¯)][Re(Z*(ωi))−Re(Z¯*)]}

where *E*[·] is the expectation operation; $Z\left(\omega_{i}\right)$ and $Z^{*}\left(\omega_{i}\right)$ signify the impedance responses at the *i*th frequency before and after a damage event, respectively; $\bar{Z}$ and ${\bar{Z}}^{*}$ indicate the mean values of those impedance responses; and $\sigma_{Z}$ and $\sigma_{Z}^{*}$ are the corresponding standard deviations. 

As another damage-sensitive feature, the RMSD index can be obtained by [23] the following:

(8) $$\mathrm{RMSD}=\sqrt{\sum_{i=1\;}^{N}{\left[Z^{*}(\omega_{i})-Z(\omega_{i})\right]}^{2}/\sum_{i=1}^{N}{\left[Z(\omega_{i})\right]}^{2}}$$

where *N* denotes the number of swept frequencies.

For distinguishing the bolt-loosening state from the healthy state, an alarming threshold known as the upper control limit (UCL) can be established using the values of the impedance features under the intact state [25,37], as follows:

(9) UCL=μ+3σ

where *μ* and *σ* are the mean and the standard deviation of the impedance feature values, respectively. The UCL determined by three standard deviations of the mean has the confidence level of 99.7%. 

##### Preload Change Detection Results

**Figure 14:**
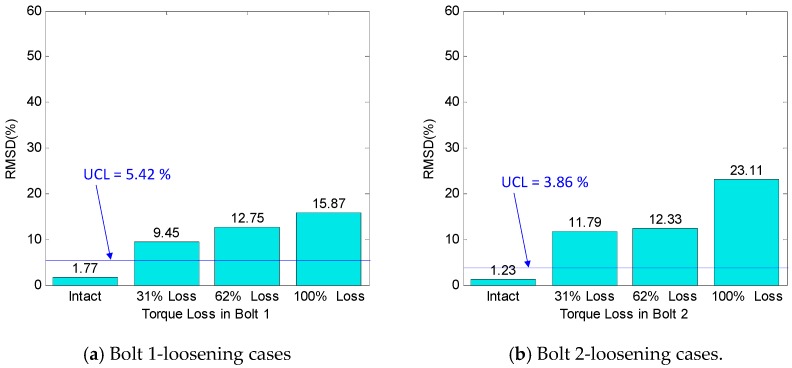

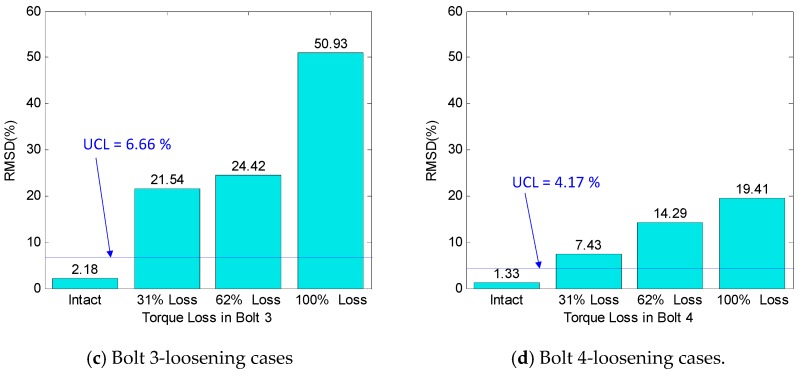
Preload change monitoring of bolted connection by root-mean-square deviation (RMSD) index.

The RMSD control chart was constructed using the impedance data in the predetermined frequency band of 10–50 kHz. Figure 14 shows the RMSD index that was plotted according to the torque-loss level for all of the loosening cases of Bolts 1–4. As observed from Figure 14a–d, the RMSD values were very small for the intact case, but became noticeable as Bolts 1–4 experienced a 31%, 62%, and 100% torque-loss. The UCL thresholds were computed to classify the bolt-loosening events. As plotted in Figure 14a–d, for all of the torque-loss events, the RMSD values were above the defined thresholds, indicating the successful detection of the preload changes.

It should be noted that the 31% torque-loss of a single bolt is equivalently corresponding to the 3.8% preload reduction in the test connection, which consists of eight bolts. This means that the impedance signatures of the PZT interface were quite sensitive to the small preload changes occurred in the bolted connection. The loosened bolts near the PZT interface (i.e., Bolts 2 and 3) were detected with higher severity estimations than those far from the interface (i.e., Bolts 1 and 4). These results confirmed that the single PZT interface was able to monitor multiple loosened bolts on the splice plate. 

**Figure 15:**
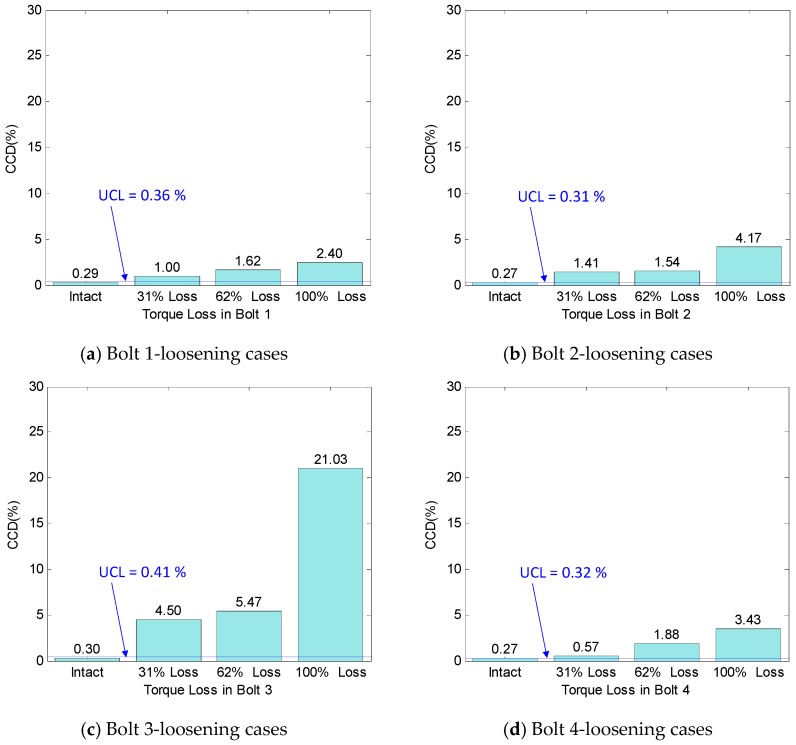
Preload change monitoring of bolted connection by correlation coefficient deviation (CCD) index.

For the comparison, the CCD control chart was also constructed by using the same impedance data, as shown in Figure 15a–d. As compared with the RMSD, the values of the CCD index were relatively smaller. It is noted from Figure 13 that the impedance signatures showed both frequency and magnitude changes under the bolt-loosening events. Thus, the RMSD approach considering both of the magnitude and frequency shifts is expected to result in higher severity estimations than the CCD approach quantifying only the frequency shift. The thresholds of the CCD index were computed and are also sketched in Figure 15a–d. Although the values of the CCD index were quite small, those for the damage cases were above the UCL thresholds, indicating the successful bolt-loosening detection.

## 5. Conclusions

In this study, the piezoelectric-based smart interface technique was developed to acquire sensitive impedance signatures from a bolted connection for bolt-loosening detection. To demonstrate the theoretical feasibility of the proposed method, a simplified EM impedance model was newly designed with the consideration of the bolt preload effect. Secondly, the EM impedance’s sensitive frequency band was numerically pre-analyzed for a bolted connection via the PZT interface technique. Finally, the impedance measurements were conducted on a lab-scaled bolted girder connection to verify the pre-analyzed sensitive frequency range and to assess the preload change in the test connection.

From the numerical and experimental observations, the following concluding remarks can be made:

(1) The PZT interface’s sensitive frequency band, predetermined by the numerical simulation, was quite consistent with that measured from the experiment.
(2) The impedance signatures obtained from the PZT interface were sensitive to the minor preload change in the bolted connection. For the tested eight-bolt connection, a 31% torque-loss of a single bolt can be detected using the PZT interface technique.
(3) A single PZT interface was able to monitor multiple loosened bolts in a connection, thus reducing the number of sensing channels for the impedance monitoring of a large bolted connection.

Future works will need to optimize the geometric size of the PZT interface so as to enhance the sensitivity of the impedance signatures and to quantitatively estimate the sensing area of the PZT interface technique. Also, there is a need to evaluate the presented method for the simultaneous loosening of multiple bolts.

## Figures and Tables

**Table 1:** Material properties of the splice plate, the PZT interface, and the bonding layer.

Parameters	PZT Interface	Splice Plate	Bonding Layer
Young’s modulus, *E* (GPa)	70	200	6
Poisson’s ratio, *υ*	0.33	0.3	0.38
Mass density, *ρ* (kg/m^3^)	2700	7850	1700
Damping loss factor, *η*	0.02	0.02	0.02

**Table 2:** Properties of the PZT-5A patch.

Parameters	Value
Elastic compliance, $s_{ijkl}^{E}$ (m^2^/N)	$\left(\begin{array}{cccccc} 16.4 & -5.74 & -7.22 & 0 & 0 & 0\\ -5.74 & 16.4 & -7.22 & 0 & 0 & 0\\ -7.22 & -7.22 & 18.8 & 0 & 0 & 0\\ 0 & 0 & 0 & 47.5 & 0 & 0\\ 0 & 0 & 0 & 0 & 47.5 & 0\\ 0 & 0 & 0 & 0 & 0 & 44.3 \end{array}\right)\times 10^{-12}$
Dielectric coupling constant, $d_{kij}$ (C/N)	$\left(\begin{array}{ccc} 0 & 0 & -171\\ 0 & 0 & -171\\ 0 & 0 & 374\\ 0 & 584 & 0\\ 584 & 0 & 0\\ 0 & 0 & 0 \end{array}\right)\times 10^{-12}$
Permittivity, $\varepsilon_{jk}^{T}$ (Farad/m)	$\left(\begin{array}{ccc} 1730 & 0 & 0\\ 0 & 1730 & 0\\ 0 & 0 & 1700 \end{array}\right)\times \left(8.854\times 10^{-12}\right)$
Mass density, *ρ* (kg/m^3^)	7750
Damping loss factor, *η*	0.0125
Dielectric loss factor, *δ*	0.015

**Table 3:** Damage cases of the finite element (FE) model.

Damage Case	Description	Value of Contact Stiffness (N/m^2^/m)	
		*k_x_ = k_y_*	*k_z_*
Intact	0% contact stiffness-loss	2.0 × 10^11^	4.0 × 10^11^
D1	12.5% contact stiffness-loss	1.75 × 10^11^	3.5 × 10^11^
D2	25% contact stiffness-loss	1.5 × 10^11^	3.0 × 10^11^
D3	37.5% contact stiffness-loss	1.25 × 10^11^	2.5 × 10^11^
D4	50% contact stiffness-loss	1.0 × 10^11^	2.0 × 10^11^

**Table 4:** Change in the peak frequencies due to contact stiffness-loss.

Damage Case	Peak Frequency (kHz)			
	*f*_1_	Δ*f*_1_ (%)	*f*_2_	Δ*f*_2_ (%)
Intact	18.05	0	34.05	0
D1	17.80	−1.39	34.00	−0.15
D2	17.50	−3.05	33.95	−0.29
D3	17.20	−4.71	33.90	−0.44
D4	16.80	−6.93	33.85	−0.59

**Table 5:** Preload change cases of bolted girder connection.

Loosened Bolt	Variation of Torque Level (Nm)
Bolt 1	Bolt 1: 160 → 110 (−31%) → 60 (−62%) → 0 (−100%); all others: 160
Bolt 2	Bolt 2: 160 → 110 (−31%) → 60 (−62%) → 0 (−100%); all others: 160
Bolt 3	Bolt 3: 160 → 110 (−31%) → 60 (−62%) → 0 (−100%); all others: 160
Bolt 4	Bolt 4: 160 → 110 (−31%) → 60 (−62%) → 0 (−100%); all others: 160
